# Surface Integrity of Glass-Ceramics by Laser-Assisted Diamond Cutting

**DOI:** 10.3390/mi16091054

**Published:** 2025-09-16

**Authors:** Jiawei Li, Fang Ji, Feifei Xu

**Affiliations:** Institute of Machinery Manufacturing Technology, China Academy of Engineering Physics, Mianyang 621900, China

**Keywords:** glass-ceramics, removal mechanism, laser-assisted diamond cutting, surface integrity

## Abstract

Glass-ceramic optical components are extensively employed in advanced optical systems. The high-hardness and low-fracture toughness of glass-ceramics make it prone to cracks and subsurface damage during conventional cutting. The laser-assisted diamond cutting method can significantly improve the nano-cutting performance of glass-ceramics by locally heating and softening the material. However, its dynamic removal mechanisms remain unclear. The coupling mechanisms between the laser thermal field and the mechanical response of the material require further investigation. This study aims to reveal the dynamic removal mechanisms of glass-ceramics under laser-assisted nanoscale cutting conditions through numerical simulations and systematic experiments. It includes a systematic analysis of the effects of laser heating on chip morphology, temperature fields, stress fields, and cutting forces using a laser-assisted nano-cutting model. Additionally, through nanoscale taper cutting experiments, this study quantifies the enhancement effect of laser power on the critical depth of no observed surface cracks (NOSC). Finally, subsurface integrity results elucidate the mechanisms through which laser assistance inhibits crack propagation. The findings will provide theoretical support for optimizing laser-assisted cutting parameters and achieving high-quality machining of glass-ceramics.

## 1. Introduction

Glass-ceramics exhibit excellent properties such as a near-zero thermal expansion coefficient and high transparency across a wide spectral range [1], which has led to their widespread application in fields such as aerospace observation and optical instruments. Glass-ceramics possess good mechanical strength and hardness, while their fracture toughness is relatively low, indicating they’re a typical hard and brittle material [2]. High-quality machining is challenging because brittle removal often occurs during machining, leading to surface and subsurface damage that affects the overall performance of the component [3].

The performance and stability of glass-ceramic optics are highly dependent on the finish quality of optical components. At present, the form accuracy of glass-ceramic optics is required to meet the specification of 1/30–1/50λ. To meet the required shape accuracy and surface quality control, glass-ceramic optical components are machined through multi-processes, including precision grinding, rapid polishing, rough polishing, and fine polishing. Precision grinding can achieve sub-micron surface accuracy, and is widely used in the manufacture of various optical materials [4]. However, grinding tends to leave severe subsurface damage, significantly limiting the improvement of the machining efficiency for glass-ceramic optical components. In addition, when it comes to special structural components such as microlens arrays and concave cones, grinding wheel interference greatly limits the application of this method.

Ultra-Precision turning Technology has gained extensive application [5], owing to its high cost-effectiveness, environmentally friendly process, superior form accuracy control capabilities, and exceptional adaptability to complex geometries [6,7]. However, its cutting performance on hard, brittle materials is constrained by critical edge degradation issues. Specifically, acute diamond tool edges incur micro-chipping, fracture, and accelerated mechanical wear [8] during machining processes. This damage mechanism fundamentally limits practical implementation in precision optics manufacturing [9]. Zhang et al. [10] observed severe micro-groove mechanical wear on diamond tools when turning RB-SiC material. Conventional diamond turning faces significant challenges in achieving ductile regime removal of fused silica, as hard, brittle materials inherently undergo brittle fracture under standard machining conditions. Scratch testing at a 30 nm cutting depth confirmed persistent brittle removal characteristics in fused silica, with subsurface cracks extending up to 200 nm [11]. This critical crack-to-cut depth ratio conclusively demonstrates the limitations of conventional SPDT for ductile processing of this optical material. At present, there is no mature technology application for the mass manufacturing of glass-ceramic optical components through diamond turning.

Laser-assisted diamond turning (LADT) guides the laser beam to pass through a transparent diamond tool to locally heat the workpiece material in the cutting region [12]. This method effectively enhances the ductile-brittle transition depth [13], facilitating ductile ductile removal of hard and brittle materials [14], and achieves a higher material removal rate compared to ultraprecision grinding while maintaining the same surface quality. The feasibility of the LADT method has been successfully demonstrated in sapphire [15], silicon [16], and tungsten carbide [17] materials before. The surface roughness achieved by LADT can reach 1–5 nm, showing its broad application prospects.

At present, there is a strong demand for high-quality machining of glass-ceramic, and there is an urgent need to improve machining efficiency and quality by turning instead of grinding. Glass-ceramic material presents an obvious decrease in hardness at elevated temperature, which has been demonstrated by Milhans et al. [18] using high-temperature nanoindentation techniques. Their results clearly demonstrated that both the hardness and elastic modulus of the material decreased significantly as the testing temperature was elevated from 25 °C to 400 °C, confirming the distinct thermal softening characteristics of this glass-ceramic. This substantial reduction in hardness reflects an enhanced plastic deformation capacity of the material at elevated temperatures, proving that LADT is a highly promising solution. However, there are still several core technical bottlenecks that need to be addressed. Firstly, the laser heating thermal field needs to be accurately controlled to achieve sufficient high-temperature softening of the material and also avoid thermal damage and high-temperature graphitization of the diamond tool [17]. Furthermore, the enhancement of nano-cutting performance under laser-assisted nano-cutting conditions for glass-ceramics is still unclear, and it is crucial to determine the critical depth of no observed surface cracks (NOSC) through taper cutting experiments. Finally, the impact of laser-assisted nano-cutting on the subsurface integrity of glass-ceramics needs to be systematically analyzed to provide guidance for the material removal depth in subsequent polishing steps.

To address the research gaps and bottlenecks in glass-ceramic machining using the LADT method, we conduct a systematic investigation of the laser heating thermal field prediction and control. A laser-assisted nano-cutting model for glass-ceramics is established, and a series of experiments is performed to elucidate the material-removal mechanisms under in-situ laser assistance. These findings provide a theoretical foundation for high-quality, high-precision machining of glass-ceramic optical components.

## 2. Laser Power Selection

Material temperature is a vital index in laser-assisted turning (In-LAT) and significantly affects machining quality. An appropriate heating temperature reduces material hardness and improves machinability, while excessive temperature induces undesired thermal damage and severe diamond tool wear. It is essential to keep the laser heating temperature within an optimal range, which mainly depends on the irradiated laser power. Numerical calculation facilitates thermal field analysis and cuts down experimental efforts; combined with thermal initial conditions and complex boundary conditions, the workpiece thermal field can be precisely calculated.

In the case of a boundary heat source, if the workpiece moves in the positive x-direction with a constant velocity $V_{\mathrm{laser}}$, the governing heat conduction equation can be defined by:

(1) $$\nabla^{2}T-\frac{\rho c_{p}}{k}V_{\mathrm{laser}}\frac{\partial T}{\partial x}=0$$

where ρ is the density in ${\mathrm{kg}/m}^{3}$, $c_{p}$ is the material specific heat capacity in J/(kg·K), *k* is the thermal conductivity in W/(m·K).

The boundary condition of the boundary laser heat can be expressed as:

(2) $$k\frac{\partial T}{\partial z}(x,y,0)=-\frac{2AP}{\pi R^{2}}e^{-\frac{2{(x}^{2}+y^{2})}{R^{2}}}$$

where −∞<x<+∞; −∞<y<+∞; z>0, where *R* is the radius of the laser spot, *P* is the energy of the laser irradiation on the material surface, and *A* is the absorptivity of the material to the laser. Since the absorption coefficient of glass-ceramics for the 1064 nm laser is high, the heat flux is simplified by assuming the laser is absorbed on the material surface, loading only as a surface heat source without considering absorption in the depth direction.

The basic equation for the analysis of heat conduction is Fourier’s law:

(3) $$q_{n}=-K_{n}\frac{\partial T}{\partial n}$$

where heat flux $q_{n}$ is the heat transfer rate in the n direction per unit area perpendicular to the direction of heat flow. ∂T/∂n (K/m) is the temperature gradient in the direction n. The $K_{n}$ (${W\cdot m}^{-1}\cdot K^{-1}$) is the thermal conductivity in the n direction.

**Figure 1:**
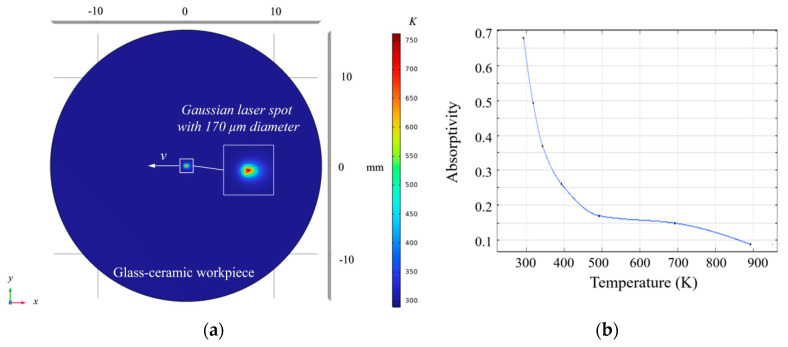
FEA simulation of the temperature field caused by the laser heating: (**a**) model dimensional setup, (**b**) temperature-dependent absorption parameters of the glass-ceramics.

The laser-induced heating temperature is a critical process parameter for LADT technology. However, accurate determination of the workpiece temperature field during LADT is impeded by the limited spatial resolution of existing thermometric devices and by the occlusion of the cutting zone by the tool. Consequently, a finite-element thermal-field model of the workpiece has been established. Considering the extremely low absorption coefficient of diamond at the working wavelength of 1064 nm and its near-ideal thermal expansion properties, the temperature variation within the diamond tool is negligible [19]. Thus, only the workpiece thermal field was calculated in the simulation model. During the simulations, the peak temperature within the workpiece was employed as a diagnostic probe to assess the evolution of the thermal field under different laser-power levels. To characterize the surface temperature distribution induced by laser irradiation at various power settings, a simplified three-dimensional transient heat-conduction model was constructed using the finite element simulation software. Because the projected shape of the laser beam is irregular and not readily amenable to analytical description, the beam was approximated by a circular spot in the finite-element model, as illustrated in Figure 1a. The laser spot in Figure 1a deviates from a Gaussian distribution owing to the laser’s high-speed scanning, forming a symmetric “comet tail”-like profile.

To improve temperature simulation accuracy, a laser temperature measurement experiment was conducted. A 1064 nm laser irradiated the workpiece surface through a diamond tool, with thermocouples around the heating point. Using a micro laser-assisted system, thermocouples, and a Φ30 × 5 mm glass-ceramic workpiece, temperatures at 2.5 mm and 4 mm from the heating point under varying laser powers were measured and compared with simulations. Temperature was recorded in three stages: preheating (0–40 s), laser heating (40–160 s), and natural cooling (160–450 s). Initial simulations deviated greatly from experiments due to ignoring the temperature—dependent absorptivity of glass-ceramic (0.68 at room temperature for 1070 nm laser). Via COMSOL parameter optimization, a nonlinear absorptivity-temperature relationship (absorptivity decreases with temperature) was obtained.

The numerical simulations employed a moving surface heat source to represent a continuous-wave 1064 nm laser (spot diameter = 170 µm) operating at power levels of 10, 20, 30, and 35 W. The laser irradiated a glass-ceramic workpiece (diameter = 30 mm, thickness = 5 mm) while scanning radially at a traverse speed of 200 mm min^−1^. The finite-element model incorporated forced convection from the cutting fluid, natural convection to ambient air (heat-transfer coefficient = 20 W·m^−2^·K^−1^), and radiative heat loss. A convective heat-transfer coefficient of 300 W·m^−2^·K^−1^ for the minimum-quantity lubrication (MQL) was adopted, following Kurgin et al. [20]. To balance computational accuracy and efficiency, mesh sizes ranging from 2 μm to 150 μm were applied on the workpiece end face. Based on experimental optimization of temperature-dependent absorption parameters of the glass-ceramics, an interpolation function was established to define the material’s absorptivity as a function of temperature, as illustrated in Figure 1b. Through experimental verification, the errors of the optimized model under different heating powers are within 7.2% at 2.5 mm from the center and within 5.5% at 4 mm from the center. These error ranges are within the acceptable limits for experimental measurements, thereby validating the rationality and reliability of the optimized temperature field model. The simulation parameters have been listed in Table 1. The physical properties of the glass-ceramics were referenced from [21].

**Figure 2:**
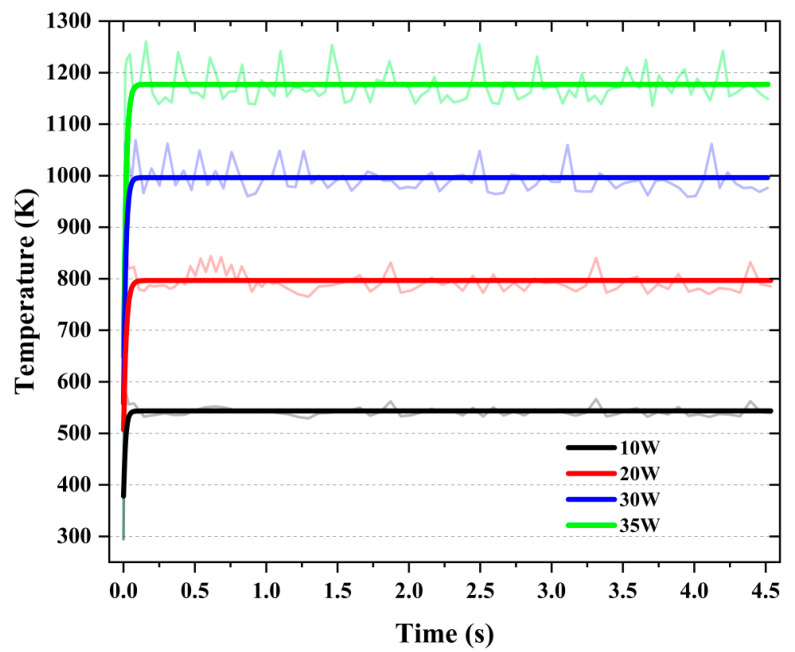
Simulated maximum temperature of the glass-ceramics workpiece under different laser power conditions.

The maximum temperatures of the workpiece under laser powers of 10 W, 20 W, 30 W, and 35 W are shown in Figure 2. Figure 2 exhibits fluctuations attributed to mesh size limitations in the finite element simulation, where the more fluctuating curve corresponds to raw data extracted from the model and the smoother one denotes the fitted version. As observed from the temperature curves in Figure 2, the workpiece temperature increases significantly with rising laser power from 10 W to 35 W. In all cases, a rapid temperature rise occurs during the initial heating stage, followed by a stabilization phase at different temperature levels. For instance, a low laser power of 10 W stabilizes around 550 K, while a high power of 35 W results in a steady-state temperature of approximately 1280 K. The higher laser power delivers greater energy density, allowing the workpiece surface to rapidly absorb heat and increase in temperature within a short time. Given that the onset of diamond graphitization is generally accepted to occur near 1000 K—with further acceleration at higher temperatures [22]—it is noteworthy that a laser power of 30 W yields a steady-state surface temperature of approximately 1000 K. At this temperature, the risk of diamond graphitization is mitigated, while the laser-induced softening of the glass-ceramics surface is maximized. This reduction in mechanical strength facilitates material removal by the diamond tool with minimal cutting force.

## 3. Experimental Setup

The NOSC depth is a key metric for evaluating the machining performance of hard and brittle materials. This work systematically examines the effect of laser in-situ heating on the NOSC depth of glass-ceramics by conducting taper cutting experiments under various laser-power conditions. Experimental validation was performed on an ultra-precision lathe using a cylindrical glass-ceramic workpiece (Ø 30 mm, 5 mm thick). To eliminate the influence of the initial surface condition, all specimens were meticulously polished to guarantee Sa < 2 nm, resulting in a near-defect-free surface. The workpiece was mounted on the spindle end face with a precision fixture offering fine adjustment. Before each cut, the fixture was calibrated in situ with an integrated micrometer to guarantee that the workpiece end surface was exactly perpendicular to the spindle axis. The experiment was carried out with a single-crystal diamond tool (rake angle −35°, clearance angle 10°, nose radius 0.5 mm) mounted in a custom tool holder that maintained a vertical tool face throughout the process.

**Figure 3:**
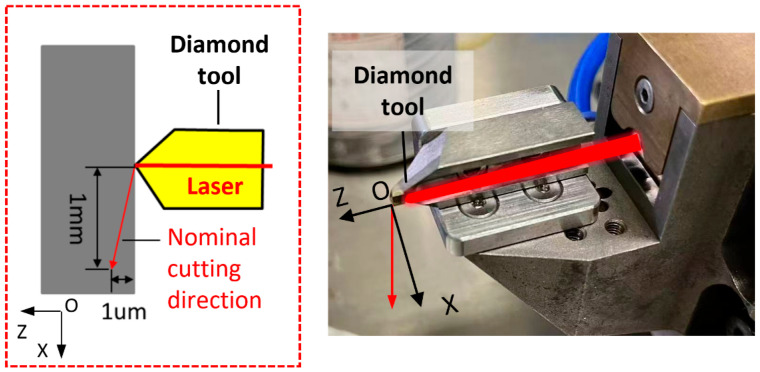
Schematic diagram of glass-ceramics taper cutting experiment.

In the experiments, laser power was set at 0 W, 10 W, 15 W, 20 W, 25 W, and 28 W. Although it was found through simulation in Section 2 that a 30 W laser is optimal, the laser used in the experiment supports a maximum laser output power of 75 W. When the laser spot is adjusted to the ideal position, the maximum laser power emitted from the tool is 28 W. Therefore, the maximum laser power set in the experiment is 28 W. The taper cutting motion in the diamond cutting experiments was achieved through precise interpolation of the machine’s *X*-axis and *Z*-axis, as shown in Figure 3, allowing a preset constant slope ratio of 1:1000. Furthermore, by rotating the spindle’s C-axis, multiple independent taper cutting tests could be performed at different angular positions while the workpiece remained in the same clamping state. To ensure the reliability of the experimental results and exclude influences from random factors, four independent taper cutting experiments were repeated for each specified laser power level, and the final analysis utilized the average values from these four repeated experiments. Specific cutting process parameter settings are detailed in Table 2.

## 4. Critical Depth of No Observed Surface Cracks

**Figure 4:**
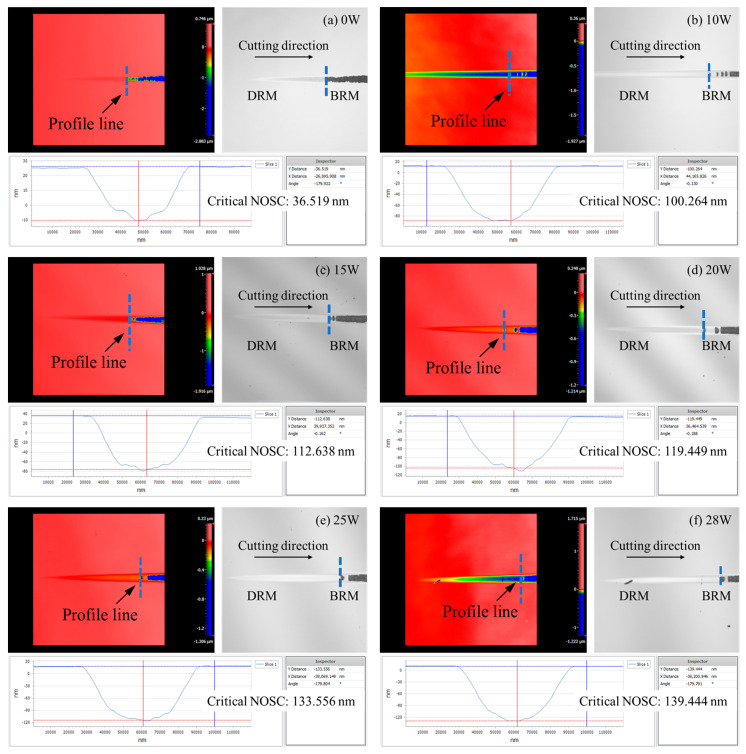
Critical NOSC depth of grass-ceramics under laser assistance with laser power of: (**a**) 0 W; (**b**) 10 W; (**c**) 15 W; (**d**) 20 W; (**e**) 25 W; (**f**) 28 W.

To assess the NOSC depth of glass-ceramics under different parameter groups, the white light interferometer was employed to measure the surface morphology after taper cutting. The experimental results indicate that laser power significantly enhanced the NOSC depth of glass-ceramics. The exact positions for extracting the cross-sectional profiles in Figure 4 are marked with blue dashed lines. Without laser assistance (0 W), the NOSC depth was only 36.519 nm (Figure 4a). When the laser power increased to 10 W, the transition depth significantly increased to 100.264 nm (Figure 4b). And at 28 W power conditions, it further increased to 139.444 nm (Figure 4f). This change indicates that the material was heated and softened by the in-situ laser heating, reducing the inter-grain bonding forces and allowing glass-ceramics to undergo plastic deformation more easily, thereby enhancing machining performance.

**Figure 5:**
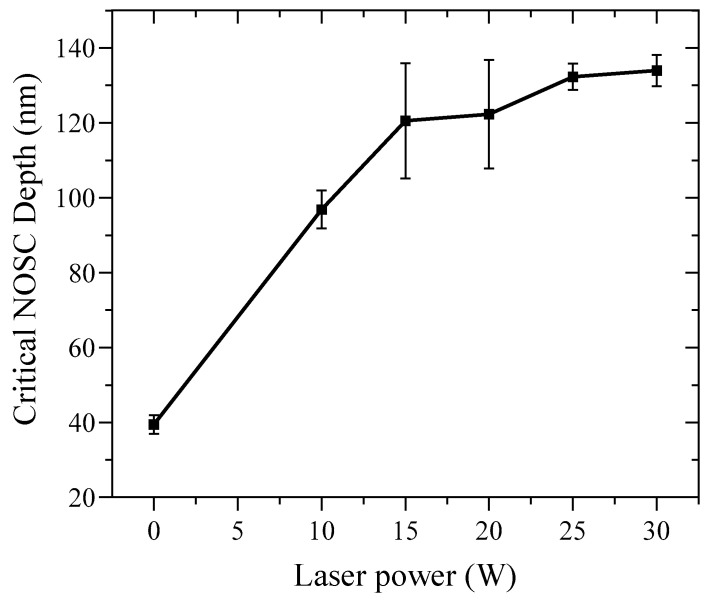
Critical NOSC depth from taper cutting experiments at different laser powers.

Based on the experimental results (Table 3), the critical NOSC depth increases with laser power, exhibiting a monotonic rise up to 20 W. Beyond this threshold, the growth asymptotically approaches saturation. The statistical distribution of NOSC depths (Figure 5) demonstrates a high degree of repeatability and consistent transition depths across the various laser-power settings.

Combining the experimental results, this study not only reveals the significant enhancement of critical NOSC depth of glass-ceramics due to laser-assisted cutting, but also provides a theoretical basis for optimizing LADT machining parameters. In practical applications, suitable power ranges and machining parameters can be selected based on the matching relationship between undeformed chip thickness and laser power to achieve high-efficiency and high-quality machining of glass-ceramics.

## 5. Surface Generation Mechanism

### 5.1. Numerical Simulation

The laser-assisted nano-cutting model was established based on the finite element analysis (FEA) to evaluate the influence of laser in-situ heating on the glass-ceramics material removal mechanism. The chip temperature distribution, cutting forces, and stress fields were systematically calculated and analyzed.

**Figure 6:**
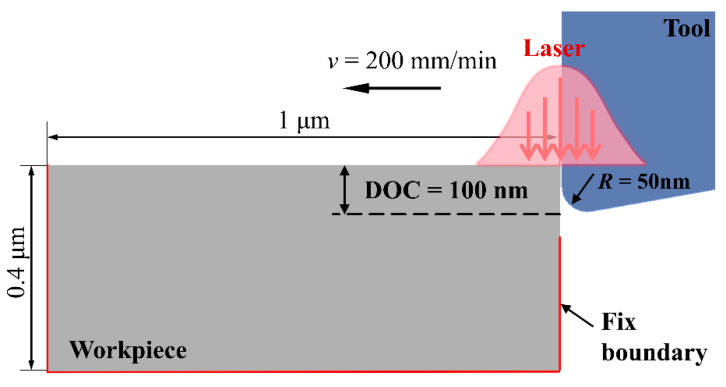
Model schematic of laser-assisted cutting of glass-ceramics.

To achieve an appropriate compromise between computational accuracy and efficiency, a two-dimensional orthogonal-cutting finite-element model was developed in this study. The geometric configuration of this model is shown in Figure 6. Laser heating was introduced via user-defined subroutines written in Fortran, which impose a time-dependent thermal load synchronized with the instantaneous position of the cutting tool, thereby reproducing the characteristics of in-situ laser heating. To maximize heating efficiency and tightly control the thermal-affected zone, the laser-spot centre was continuously positioned at the tool tip. Accordingly, dynamic heat-flux boundary conditions were applied to the workpiece surface, with the heat-flux density calculated from the Gaussian-beam energy distribution for a beam of 170 µm diameter.

The geometric dimensions and cutting parameters employed in the numerical model were chosen to replicate the experimental conditions. The workpiece is defined with macroscopic dimensions of L = 1.0 µm (length) and H = 0.4 µm (height). The cutting tool is made of monocrystalline diamond; owing to its exceptionally high hardness and elastic modulus, the tool is modeled as an analytically rigid body. The tool geometry is specified as a rake angle of 0°, a clearance angle of 10°, and, to account for the finite sharpness of a real diamond edge, an edge radius of 50 nm is introduced. Cutting conditions identical to those used in the experiments are imposed: a nominal depth of cut of 100 nm and a cutting speed of 200 mm min^−1^. During the simulation, the tool translates uniformly in the negative X-direction. A complete list of the model parameters is provided in Table 4.

#### 5.1.1. Thermal Field

**Figure 7:**
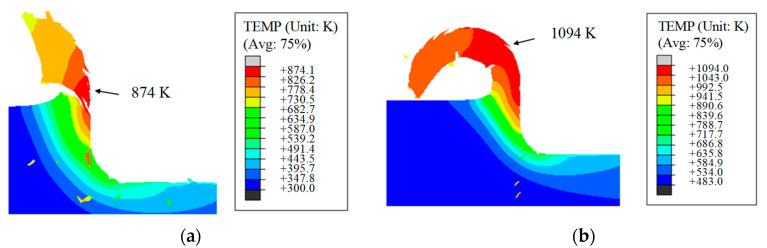
Workpiece thermal field in the process of: (**a**) conventional nano-cutting; (**b**) laser-assisted nano-cutting.

Using a two-dimensional orthogonal cutting model, the temperature field of the glass-ceramic workpiece and cutting chips was investigated under conventional and 28 W laser-assisted cutting conditions. Figure 7 presents the comparison at a cutting distance of 0.87 µm. The simulation results clearly show that laser assistance markedly modifies the cutting process. In conventional nano-cutting (Figure 7a), material removal occurs in brittle mode, producing a segmented, discontinuous chip. The temperature rise in the workpiece is dominated by tool–workpiece friction, and the elevated-temperature region is confined to the primary and secondary deformation zones, reaching a maximum of 874 K. When applying laser-assisted cutting (Figure 7b), the laser energy fundamentally changes the material’s removal behavior. The chip becomes noticeably more curled and continuous, which indicates a transition from brittle fracture to ductile removal mode. Under the 28 W laser power, the laser heating becomes the dominant thermal source, significantly raising the temperature in the cutting zone. The maximum temperature reaches 1094 K, and this localized softening of the glass-ceramics directly contributes to the improved ductility and smoother chip formation.

#### 5.1.2. Cutting Force

Cutting force is a fundamental physical quantity in ultra-precision machining and serves as a critical metric for assessing tool load, predicting wear progression, and optimizing machining parameters. In the FEA simulations, the forces acting on the tool were recorded continuously with a temporal resolution of 1 ns. To extract the primary trend of cutting-force variations and suppress high-frequency numerical noise, the raw data were post-processed using a low-pass filter based on the Fast Fourier Transform (FFT), with a cutoff frequency set at 1.25 MHz.

**Figure 8:**
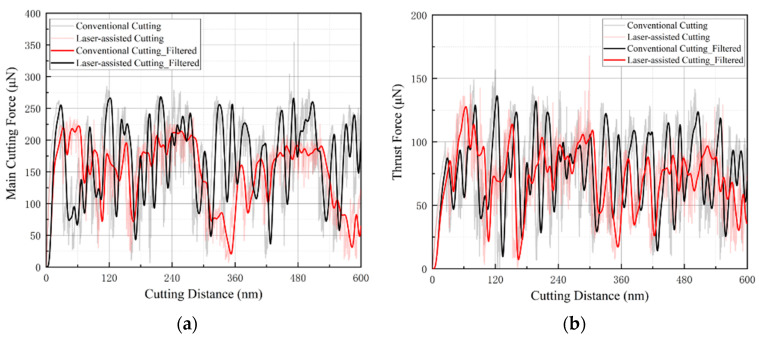
Comparison of nano-cutting forces with and without laser assistance: (**a**) main cutting force; (**b**) normal cutting force.

Figure 8 illustrates the variation of the cutting-force components as a function of cutting distance, both with and without laser heating assistance. Figure 8a shows the main (tangential) cutting force, which is aligned with the cutting-speed direction, whereas Figure 8b presents the normal (thrust) force acting perpendicular to the machined surface. The curves clearly indicate that laser heating softens the glass-ceramic material, thereby reducing its cutting resistance.

To quantify this effect, the filtered force signals were averaged over the entire cutting distance. For the main force, the mean value without laser assistance is ≈195 µN, which drops to ≈160 µN when laser heating is applied—a reduction of roughly 17.9%. For the normal force, the average decreases from ≈95 µN in the conventional case to ≈85 µN under laser-assisted conditions, corresponding to a reduction of about 10.5%. These results demonstrate that LADT can markedly reduce cutting forces, confirming that laser heating induces a softening effect of glass-ceramics, thereby mitigating tool wear and extending tool life.

### 5.2. Subsurface Crack

**Figure 9:**
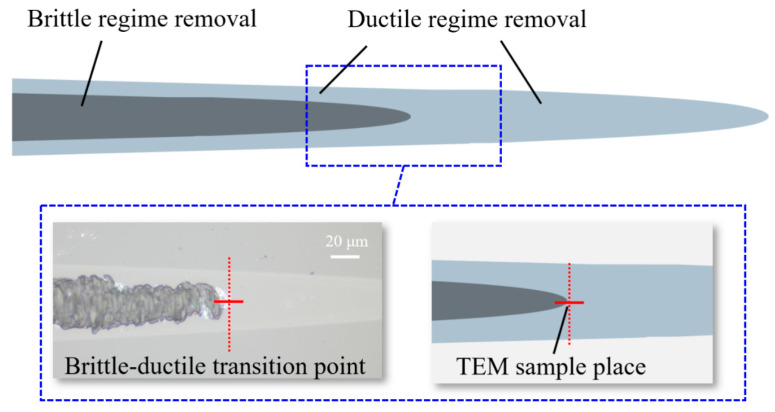
Schematic diagram of TEM sample position.

To assess the subsurface quality of the machined glass-ceramic workpiece, thin samples were harvested from taper cutting representative zones for transmission electron microscopy (TEM) analysis. Using a focused ion-beam (FIB) system, an 8 × 5 × 1 µm sample was lifted from the cross-section of the ductile-brittle transition region and subsequently thinned to a uniform thickness of 50–70 nm to enable detailed TEM characterization of sub-surface characteristics. The ductile–brittle transition zone of the material is defined by the simultaneous presence of surface crack and ductile cutting features. Consequently, a localized sub-surface region of approximately 3 µm surrounding the transition point was selected for sample preparation, allowing high-resolution observation of subsurface cracks in the glass-ceramic. The sampling location is indicated in Figure 9.

**Figure 10:**
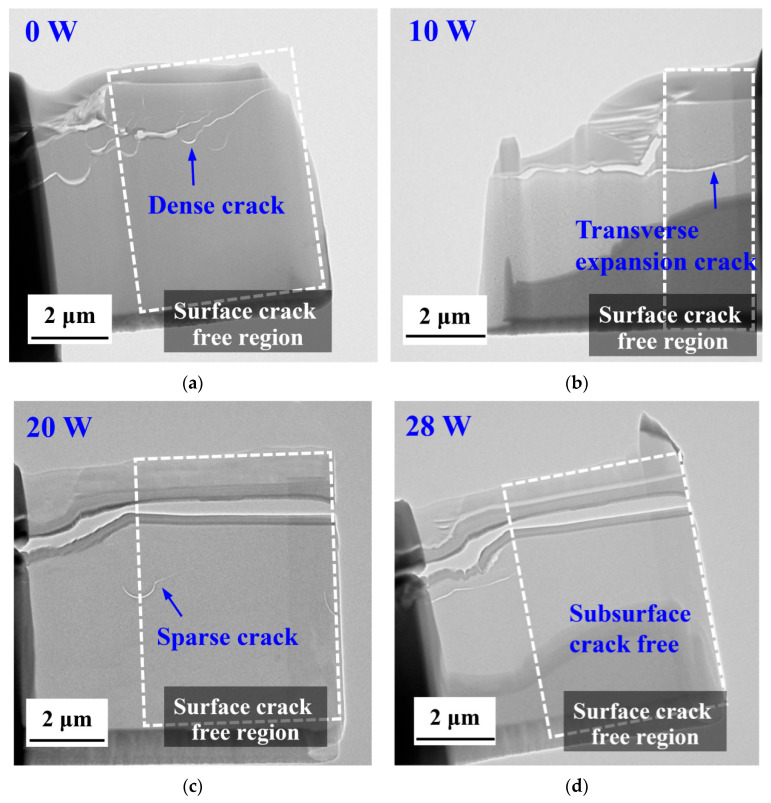
Comparison of subsurface damage of glass-ceramics by: (**a**) traditional Cutting; (**b**) 10 W laser-assisted nano cutting; (**c**) 20 W laser-assisted nano cutting; (**d**) 28 W laser-assisted nano cutting.

The exact positions for extracting the cross-sectional profiles in Figure 9 are marked with red dashed lines. Subsurface damage in glass-ceramic workpieces—manifested by crack initiation and propagation—can degrade the component’s functional performance. Among the process parameters, laser power is the dominant factor that governs both the extent of crack propagation and the depth of subsurface damage. Accordingly, this subsection presents a systematic investigation of specimens machined at laser powers of 0 W, 10 W, 20 W, and 28 W (Figure 10). Surface crack-free regions are framed in the figure, permitting a direct comparison of crack-propagation length and subsurface-damage depth under the different laser-power conditions.

Under conventional nano-cutting (laser power = 0 W), the subsurface region of the glass-ceramic exhibits a dense network of interlaced cracks both in the brittle removal regime and the surface crack-free region, extending to a depth of approximately 2 µm, as shown in Figure 10a. This observation clearly demonstrates the pronounced brittleness of the glass-ceramic under traditional cutting: cracks readily propagate along grain boundaries or pre-existing defects, forming a network-like pattern.

The introduction of laser energy locally thermal softens the material, thereby diminishing the driving force for crack propagation. The emergence of transversely oriented cracks indicates a change in the propagation direction. Laser-induced thermal stresses, superimposed on the pre-existing mechanical stresses, generate specific directional stresses within the glass-ceramic. Consequently, thermal softening reduces the stress concentration at crack tips, suppresses longitudinal branching, and promotes lateral crack growth as illustrated in Figure 10b. Simultaneously, residual thermal stresses combined with mechanical loading drive deeper crack penetration.

When the laser power is raised to 20 W (relative to the 10 W condition), the subsurface cracks in the surface crack-free region become sparse, with a reduced depth of approximately 3.8 µm (Figure 10c). Further increase of the laser power expands the absorbed energy, enlarging the thermal influence zone. This enlarges the plastic-deformation region during machining, reduces the proportion of brittle fracture, and curtails extensive crack propagation, leaving only a few isolated cracks.

No subsurface cracks are observed in the surface crack-free region for the 28 W laser-assisted nano cutting, as shown in Figure 10d. At this power level, the laser markedly increases the material’s fracture toughness while decreasing its hardness; the lower cutting force diminishes the cutting stress, and pronounced thermal softening enhances the material’s machinability. These combined effects prevent crack initiation and propagation, thereby virtually eliminating subsurface cracks.

### 5.3. Subsurface Integrity

Subsurface damage encompasses more than the mere presence or absence of cracks; it also involves internal microstructural alterations—such as lattice distortion, grain refinement, and microcrack initiation—that directly affect critical performance metrics, including fatigue life, wear resistance, and corrosion resistance. Consequently, a comprehensive investigation of the morphology and mechanisms of subsurface damage across various laser-power levels is required to systematically characterize the specific damage forms under each condition and to elucidate their underlying physicochemical mechanisms, thereby providing robust scientific evidence for practical engineering applications.

**Figure 11:**
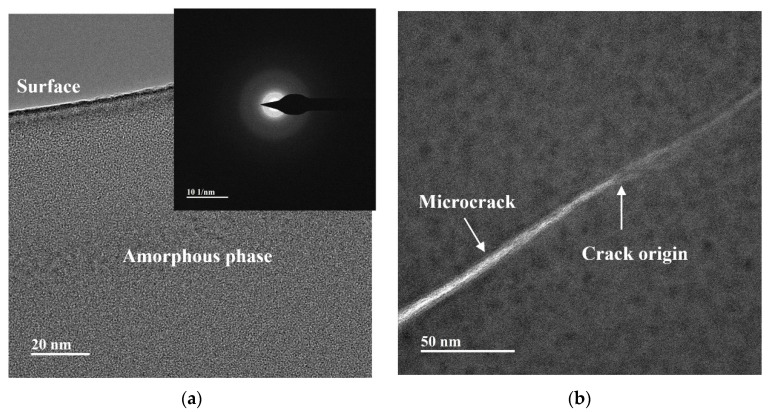
Subsurface damage of glass-ceramics under conventional nano-cutting: (**a**) near surface region; (**b**) subsurface crack region.

**Figure 12:**
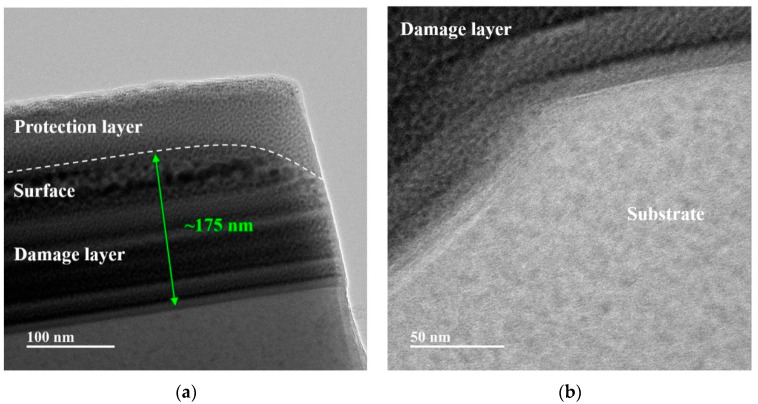
Subsurface damage of glass-ceramics under 28 W laser-assisted nano-cutting: (**a**) near surface region; (**b**) substrate region.

The subsurface damage induced by taper cutting of glass-ceramics, both without and with laser assistance, is illustrated in Figure 11 and Figure 12, respectively. In conventional nano-cutting (Figure 11a), the dominant damage mode in the near-surface region is the formation of a distinct amorphous layer. This layer originates from intense mechanical shear that disrupts the ordered β-SiO_2_ crystal lattice, yielding a disordered amorphous structure. Such a phenomenon directly reflects brittle removal, because the material lacks thermal softening and cannot accommodate appreciable ductile deformation. This observation is consistent with the shallow critical NOSC depth of 36.5 nm measured after conventional taper cutting, indicating that only an extremely thin surface region may undergo limited plastic deformation.

In contrast, the near-surface region of 28 W laser-assisted nano-cutting (Figure 12a) exhibits a uniform plastic-damage layer. Under shear stress, the crystals preferentially undergo orderly slip rather than fracture, producing a shallow plastic layer of approximately 200 nm thickness that can be readily removed by subsequent polishing. This observation is consistent with the increased critical NOSC depth observed under the 28-W laser taper cutting condition, confirming the enhanced plastic-removal capability.

The subsurface damages of glass-ceramics present diverse characteristics after taper cutting with and without laser assistance. In conventional nano-cutting (Figure 11b), the subsurface crack region contains dense microcracks and crack initiation sites, forming a “source-branch-network” structure. As a typical hard and brittle material, glass-ceramics cannot relieve internal stress through plastic deformation during conventional cutting. Stress concentration triggers crack initiation, and cracks propagate randomly along grain boundaries or defects to a depth of approximately 2 μm (consistent with Figure 10a). These network-like cracks penetrate the subsurface, reducing mechanical strength and optical performance, and require extensive polishing to remove defects. This is consistent with the observation of numerous subsurface cracks in 0 W laser-cut samples, reflecting a typical brittle removal feature.

In 28 W laser-assisted nano-cutting (Figure 12b), there are no subsurface cracks in the subsurface region, and a clear boundary exists between the plastic damage layer and the undamaged substrate. Plastic deformation dissipates most mechanical stress, preventing stress transfer to the substrate and thus inhibiting crack initiation and propagation. As a result, the substrate remains intact without structural damage, preserving the material’s original mechanical and optical properties. The distinct damage-substrate boundary avoids deep subsurface damage, which is consistent with Figure 10d, verifying effective crack suppression. The effect of lateral crack propagation and the suppression of subsurface cracks shows a clear qualitative change. As the laser power increases, on one hand, subsurface cracks, even in the brittle fracture area, are less likely to exhibit fracture characteristics. Moreover, even if cracks occur, their propagation trend is significantly suppressed.

In traditional diamond cutting, most of the cutting force, or the energy introduced by the tool, is released through brittle fracture. After processing, there is very little evidence of plastic damage or deformation in the subsurface. However, due to the heating effect of the laser, the material’s ability for plastic flow and deformation is significantly enhanced. At this point, most of the energy introduced by the tool is converted into plastic deformation of the material, leading to the formation of a larger plastic deformation damage layer. This is the main change in the material removal mechanism.

Conventional nanoscale cutting of glass-ceramics induces damage that is dominated by brittle fracture, manifested as an amorphous layer on the near-surface and deep subsurface cracks. This damage markedly degrades surface integrity and substantially raises the cost of subsequent polishing. In contrast, 28-W laser-assisted nanoscale cutting converts the material-removal mechanism to a ductile regime, producing only a thin, removable plastically-deformed layer confined to the near-surface region while suppressing the formation of subsurface cracks. Consequently, surface quality is significantly enhanced and polishing requirements are reduced, rendering this approach a reliable method for high-quality machining of glass-ceramic optical components.

## 6. Conclusions

This study systematically investigates the dynamic material-removal mechanisms of glass-ceramics under laser-assisted nanocutting conditions by integrating numerical simulations with experimental measurements. The main conclusions are as follows:

(1) Laser-heating simulations reveal that an optimal laser power of 30 W raises the temperature of the glass-ceramic to approximately 1000 K.
(2) Nano-cutting simulations indicate that in-situ laser heating markedly improves cutting performance: the average main cutting force decreases by ~17.9%, and the average normal force drops by ~10.5%.
(3) Taper-cutting experiments show that at a laser power of 28 W, the critical NOSC depth reaches 139.44 nm, representing a 281.84% increase compared with conventional cutting, thereby providing critical guidance for process-parameter optimization.
(4) TEM subsurface-damage analysis demonstrates that laser-assisted cutting effectively suppresses crack propagation. At 10 W, subsurface cracks are significantly reduced; at 28 W, no cracks are observed in the crack-free removal zone, and surface integrity improves with increasing laser power. Although a plastic damage layer of approximately 175 nm remains, it can be readily eliminated by subsequent polishing.

Collectively, these results furnish a theoretical foundation for optimizing LADT parameters, confirming the feasibility of high-quality machining of glass-ceramics by the LADT method.

## Figures and Tables

**Table 1:** Thermal field simulation parameters.

Parameters	Values
Workpiece Material	Glass-ceramics
Laser Power (P)	10, 20, 30, 35 W
Laser Beam Diameter	170 μm
Scanning Speed	200 mm/min
Absorptivity	Temp-dependent
Material Emissivity (ε)	0.62

**Table 2:** Parameters for taper cutting experiments.

Parameters	Values/Descriptions
Tool Parameters	−35° rake angle, 10° clearance angle, R0.5 mm
Cutting Speed	200 mm/min
Laser Power	0 W, 10 W, 15 W, 20 W, 25 W, 28 W
Sample Size	Φ30 × 5 mm
Slope Ratio	1:1000

**Table 3:** Experimental data of ductile-brittle transition depth as a function of laser power.

Power (W)	Experimental Results1 (nm)	Experimental Results2 (nm)	Experimental Results3 (nm)	Experimental Results4 (nm)
0	36.519	41.197	41.825	38.321
10	93.14	102.12	91.91	100.264
15	112.638	111.356	143.609	114.651
20	119.449	143.437	112.127	114.192
25	133.556	131.092	136.456	128.151
28	129.93	139.444	134.945	131.635

**Table 4:** Simulation parameters for the laser-assisted cutting finite element model.

Parameters	Values
Workpiece Material	Glass-ceramics
Cutting Depth	100 nm
Tool Edge Radius	50 nm
Cutting Speed	200 mm/min
Minimum Grid Cell Size	5 nm
Laser Power (P)	0 W, 28 W
Laser Beam Diameter	170 μm
Material Emissivity (ε)	0.62
Convection Thermal Conductivity Coefficient (h)	300 W/(m^2^·K)

## Data Availability

The original contributions presented in this study are included in the article. Further inquiries can be directed to the corresponding author.

## References

[1] Casasola R., Rincón J.M., Romero M. (2012). Glass–ceramic glazes for ceramic tiles: A review. J. Mater. Sci..

[2] Serbena F.C., Mathias I., Foerster C.E., Zanotto E.D. (2015). Crystallization toughening of a model glass-ceramics. Acta Mater..

[3] Huang H., Li X., Mu D., Lawn B.R. (2021). Science and art of ductile grinding of brittle solids. Int. J. Mach. Tools Manuf..

[4] Zhang X., Hu H., Wang X., Luo X., Zhang G., Zhao W., Wang X., Liu Z., Xiong L., Qi E. (2022). Challenges and strategies in high-accuracy manufacturing of the world’s largest SiC aspheric mirror. Light Sci. Appl..

[5] Brinksmeier E., Gläbe R., Osmer J. (2006). Ultra-Precision Diamond Cutting of Steel Molds. CIRP Ann..

[6] Hashimoto T., Yan J. (2025). Effect of System Dynamics on Surface Topography in Fast Tool Servo-Based Diamond Turning of Microlens Arrays. Nanomanuf. Metrol..

[7] Wu K., Zhang J., Zheng Z., Li Z., Ding P., Liu J., Wang J. (2024). Fabrication of Micro/Nanostructured Copper Fibers by Vibration Cutting for Felt-Based Freshwater Purification. Nanomanuf. Metrol..

[8] Kupczyk M., Jozwiak K., Cieszkowski P., Libuda P. (2007). Influence of laser heating on adhesion of CVD coatings to cutting edges. Surf. Coat. Technol..

[9] Wang J.P., Zhang G.Q., Chen N., Zhou M.H., Chen Y.B. (2021). A review of tool wear mechanism and suppression method in diamond turning of ferrous materials. Int. J. Adv. Manuf. Technol..

[10] Zhang Z., Yan J., Kuriyagawa T. (2011). Study on tool wear characteristics in diamond turning of reaction-bonded silicon carbide. Int. J. Adv. Manuf. Technol..

[11] An Q.L., Ming W., Ming C. (2015). Experimental Investigation on Cutting Characteristics in Nanometric Plunge-Cutting of BK7 and Fused Silica Glasses. Materials.

[12] You K.Y., Liu G.Y., Wang W., Fang F. (2023). Laser assisted diamond turning of silicon freeform surface. J. Mater. Process. Technol..

[13] Chen X., Liu C., Ke J., Zhang J., Shu X., Xu J. (2020). Subsurface damage and phase transformation in laser-assisted nanometric cutting of single crystal silicon. Mater. Des..

[14] Mohammadi H., Patten J.A. (2016). Laser Augmented Diamond Drilling: A New Technique to Drill Hard and Brittle Materials. Procedia Manuf..

[15] Langan S.M., Ravindra D., Mann A.B. (2018). Mitigation of damage during surface finishing of sapphire using laser-assisted machining. Precis. Eng..

[16] Ravindra D., Ghantasala M.K., Patten J.A. (2012). Ductile mode material removal and high pressure phase transformation in silicon during micro-laser assisted machining. Precis. Eng..

[17] You K.Y., Fang F.Z., Yan G.P. (2021). Surface generation of tungsten carbide in laser-assisted diamond turning. Int. J. Mach. Tools Manuf..

[18] Milhans J., Li D.S., Khaleel M., Sun X., Al-Haik M.S., Harris A., Garmestani H. (2011). Mechanical properties of solid oxide fuel cell glass-ceramic seal at high temperatures. J. Power Sources.

[19] Yilbas B.S., Akhtar S.S. (2011). Laser Cutting of Alloy Steel: Three-Dimensional Modeling of Temperature and Stress Fields. Mater. Manuf. Process..

[20] Kurgin S., Dasch J.M., Simon D.L., Barber G.C., Zou Q. (2012). Evaluation of the convective heat transfer coefficient for minimum quantity lubrication (MQL). Ind. Lubr. Tribol..

[21] Cui R., Zhou L., Ren Q., Li L., Li W. (2025). Optimizing porous glass-ceramics fabricated from coal gasification fine slag: Effects of ash composition on structure and properties. Constr. Build. Mater..

[22] Fedoseev D.V., Vnukov S.P., Bukhovets V.L., Anikin B.A. (1986). Surface graphitization of diamond at high temperatures. Surf. Coat. Technol..

